# Cytotoxicity Effect of Quinoin, Type 1 Ribosome-Inactivating Protein from Quinoa Seeds, on Glioblastoma Cells

**DOI:** 10.3390/toxins13100684

**Published:** 2021-09-25

**Authors:** Rossella Rotondo, Sara Ragucci, Salvatore Castaldo, Maria Antonietta Oliva, Nicola Landi, Paolo V. Pedone, Antonietta Arcella, Antimo Di Maro

**Affiliations:** 1 INM IRCCS Istituto Neurologico Mediterraneo NEUROMED, Via Atinense 18, 86077 Pozzilli, Italy; rossellaross1988@gmail.com (R.R.); castaldosal90@gmail.com (S.C.); mariaantonietta.oliva@neuromed.it (M.A.O.); 2 Department of Environmental, Biological and Pharmaceutical Sciences and Technologies (DiSTABiF), University of Campania “Luigi Vanvitelli”, Via Vivaldi 43, 81100 Caserta, Italy; sara.ragucci@unicampania.it (S.R.); nicola.landi@unicampania.it (N.L.); paolovincenzo.pedone@unicampania.it (P.V.P.); antimo.dimaro@unicampania.it (A.D.M.)

**Keywords:** patient-derived glioblastoma cell lines, *Chenopodium quinoa* wild, ribosome-inactivating proteins, quinoin, temozolomide

## Abstract

Ribosome-inactivating proteins (RIPs) are found in several edible plants and are well characterized. Many studies highlight their use in cancer therapy, alone or as immunoconjugates, linked to monoclonal antibodies directed against target cancer cells. In this context, we investigate the cytotoxicity of quinoin, a novel type 1 RIP from quinoa seeds, on human continuous and primary glioblastoma cell lines. The cytotoxic effect of quinoin was assayed on human continuous glioblastoma U87Mg cells. Moreover, considering that common conventional glioblastoma multiforme (GBM) cell lines are genetically different from the tumors from which they derive, the cytotoxicity of quinoin was subsequently tested towards primary cells NULU and ZAR (two cell lines established from patients’ gliomas), also in combination with the chemotherapeutic agent temozolomide (TMZ), currently used in glioblastoma treatment. The present study demonstrated that quinoin (2.5 and 5.0 nM) strongly reduced glioblastoma cells’ growth. The mechanisms responsible for the inhibitory action of quinoin are different in the tested primary cell lines, reproducing the heterogeneous response of glioblastoma cells. Interestingly, primary cells treated with quinoin in combination with TMZ were more sensitive to the treatment. Overall, our data highlight that quinoin could represent a novel tool for glioblastoma therapy and a possible adjuvant for the treatment of the disease in combination with TMZ, alone or as possible immunoconjugates/nanoconstructs.

## 1. Introduction

Ribosome-inactivating proteins (RIPs) are a group of toxins essentially retrieved in flowering plants [1]. These toxins are enzymes (N-glycosylase; EC: 3.2.2.22) able to remove a single adenine (A4324 in rat) located at a universally conserved stem and loop sequence on the large rRNA, known as the α-sarcin-ricin loop (SRL) [2]. The loss of this specific adenine causes conformational changes in the SRL structure, after which the EF-G (in prokaryotes) and eEF-2 (in eukaryotes) elongation factors are unable to interact with ribosomes during mRNA-tRNA translocation, blocking translocation during protein synthesis [3].

These enzymes are classically grouped into type 1 and type 2 RIPs based on the absence or presence of a quaternary structure. Indeed, type 1 RIPs are monomeric proteins (~30-kDa) with N-glycosylase activity while type 2 RIPs are dimeric proteins (~60-kDa) consisting of an enzymatic A-chain homologous to type 1 RIPs, linked through a disulphide bond to a B-chain with lectin properties [4]. Moreover, tetrameric protein types (A-B)2 reported in the *Sambucus* genus belonging to the family *Adoxaceae* [5] or proteolytic-activated enzymes retrieved in cereals, synthesized as inactive precursors [6], were found.

RIPs are isolated in different amounts from several plant tissues [7] and are identified in many orders belonging to angiosperms but not in gymnosperms [8,9]. Their physiological function in plants is still unknown, although it is associated with defense roles against herbivores, insects, fungi, and viruses [10]. This possible biological function is strengthened by the fact that several RIPs also have the ability to remove adenines from other substrates, such as RNAs and DNAs (‘adenine polynucleotide glycosylases’ activity), or have the capacity to cleave the phosphodiester bond (DNase activity [11]), which would amplify this function.

Research on RIPs had a great boost due to the potential biotechnological applications. In medicine, they are considered therapeutic agents against infected/tumor cells, due to the possible conjugation of type 1 or A-chain RIPs with antibodies (immunotoxins) or other carriers (peptides, specific proteins, or nanomaterials [12]) to obtain chimeric proteins able to direct these conjugates against specific targets [13,14]. In agriculture, RIPs could be employed as bio-pesticides to improve the resistance of cultivated plants towards insect, fungi, or viruses [10].

In acellular systems (*in vitro* translation), type 1 and type 2 RIPs display a similar toxicity, while in cellular systems, type 2 RIPs show higher toxicity (IC_50_ 0.0003–1.7 nM on Hela cells) with respect to type 1 (IC_50_ 170–3300 nM on Hela cells). In particular, the higher toxicity of type 2 RIPs is justified by the presence of the lectinic domain (B-chain), which possesses a strong affinity for sugar moieties on the cell surface, facilitating toxin entry into the cell [15]. Nevertheless, although less toxic, type 1 RIPs have a selective toxicity towards different cell lines, for which they could be potential drugs with clinical significance [15,16,17]. Moreover, RIPs cytotoxicity is correlated with the intracellular fate, considering the (i) expression of different types of ligands/receptors, (ii) cell surface and membrane composition (iii) routing of RIP-ligand complexes among different compartments, and (iv) availability of various pathways for transport of the A-chain into the cytosol [13].

In addition, type 1 RIPs, such as trichosanthin from *Trichosanthes kirilowii* [18,19] and saporin from *Saponaria officinalis* [20,21], display remarkable cytotoxicity against glioblastoma cell lines, which increases by linking them to specific conjugates [22]. This cytotoxicity is of interest, considering that glioblastoma is a highly aggressive brain tumor, in which malignant cells escape apoptosis by being resistant to radiotherapy and chemotherapy and unresponsive to drugs by rapidly inactivating or reducing intracellular drug concentrations or increasing the rate of DNA repair [23].

Recently, our group isolated and characterized a novel type 1 RIP from quinoa seeds, named quinoin, that displays cytotoxicity towards BJ-5ta (human fibroblasts) and HaCaT (human keratinocytes) in a dose- and time-dependent manner. Moreover, quinoin also exhibits a remarkable melting temperature (Tm ~ 68.2 °C), thermostability, and partial resistance proteolysis to cleavage [24]. These properties are of interest considering the possible use of quinoin as a natural drug alone or as an adjuvant to kill specific cells [25,26]. In this context, due to the great potential of quinoin as a toxin, we decided to test its cytotoxicity on glioblastoma cells.

Glioblastoma multiforme (GBM) is the most aggressive malignant primary brain tumor in humans, which remains incurable in most cases despite significant advances in therapy strategies [27]. GBM represents ~20% of all brain tumors and ~50% of all gliomas, being characterized by high proliferation, infiltration, and invasion, causing an objective difficulty to locally control GBM using radiotherapy or surgical excision [28]. Despite the progress in the field of neurosurgery and related treatment strategies, prognosis remains poor in most cases, with a median survival of ~14 months, due to therapeutic resistance and tumor recurrence after surgical removal, as well as tumor heterogeneity [28]. Currently, the standard GBM treatment includes maximum surgical excision, radiotherapy, and chemotherapy with temozolomide (TMZ), also known as temodal. The latter improves overall survival by ~2.5 months with respect to radiation alone, although it does not provide effective treatment of glioblastoma disease [28,29]. Therefore, we analyzed quinoin’s effects on continuous U87Mg or primary NULU and ZAR glioblastoma cell lines, focusing our attention on the latter, whose heterogeneity reproduces the parental tumor from which it is derived [30]. Moreover, considering the pharmaco-resistant mechanisms of both tested primary cell lines to the alkylating agent TMZ [30], we verified the synergistic cytotoxic effect of quinoin in the presence of temodal on both NULU and ZAR cell lines.

On the other hand, some important limitations, such as blood-brain barrier impermeability, low rate of cell degeneration, inflammatory response, and activation of compensatory mechanisms, limit the use of RIPs alone [13]. Nevertheless, many RIPs-conjugates are used in cancer gene therapy considering their possible use as weapons against cancer cells. Indeed, several immunotoxins [17] or nanoconstructs [12] were obtained to make these toxins selective. Finally, RIPs-based toxins (chimeric molecules) have been designed as molecules in which the toxic domains are linked to selective tumor-targeting domains for cancer therapy.

A clear potential of this strategy is given by saporin-6, a type 1 RIP isolated from *Saponaria officinalis* seeds. This protein, similar to quinoin [31], is very used in several conjugates in neuroscience as a convenient tool to induce highly selective degeneration of a desired cell subpopulation. Indeed, saporin-based toxins, inducing selective cell death, are one of the approaches used to study (i) neurodegenerative diseases, (ii) the functions of certain cell subpopulations in the brain, and (iii) the development of alternative therapies [21].

In this scenario, considering the thermal stability and the resistance to proteolysis [24] of quinoin as well as its similarity to saporin-6, data reported in this work are a starting point for the possible use of quinoin as a novel therapeutic tool for current GBM treatment or as a novel tool in neuroscience.

## 2. Results

### 2.1. Quinoin Isolation

Quinoin was purified from the seeds of *C. quinoa* as previously reported [24]. The homogeneity of quinoin was achieved by both SDS-PAGE and RP-HPLC analysis (Figure S1) [32].

### 2.2. Inhibiting Effect of Quinoin on Cell Growth and Viability of Glioblastoma U87Mg and Patient-Derived Cell Lines NULU and ZAR

**Figure 1:**
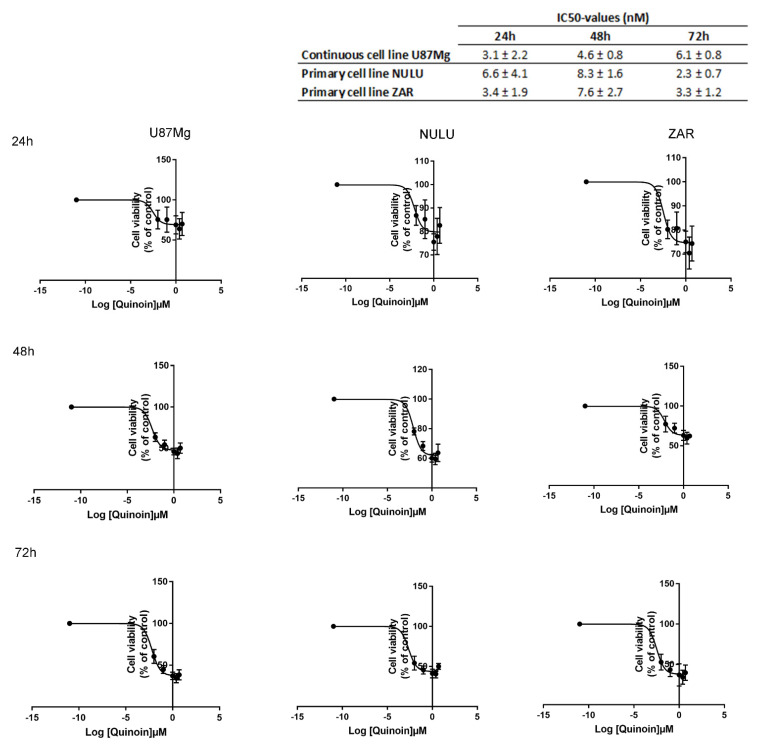
IC_50_ values estimation. IC_50_ values of U87Mg cells and two primary glioblastoma cell lines NULU and ZAR after 24, 48, and 72 h of incubation with quinoin using concentrations of 0.01, 0.1, 1.0, 2.5, and 5.0 μM. The control was assumed as part of the dose–response curve, considering it as a very low concentration (10^−11^ µM). Data were processed using GraphPad Prism and data are reported as Mean ± SD.

The inhibiting effect of quinoin occurs at a very low dose. This toxin is considered highly cytotoxic on both the U87Mg glioblastoma continuous cell line and primary cell lines NULU and ZAR as evidenced by the very low IC_50_ value (~5.0 nM). IC_50_ is the evaluation of the half-maximum inhibitory concentration of a substance and indicates the power of a drug to inhibit a specific biological or biochemical function by 50%. As reported in Figure 1, the IC_50_ values of GBM continuous and primary cells for quinoin did not exhibit a time dependence and the toxicity curves reached a plateau at high tested doses of the toxin. Similarly, what is reported in the breast cancer cell line MCF7 and glioblastoma cell line U87-Mg, type-II RIP Riproximin showed a recovery/resistance following longer exposure periods. Therefore, we can explain this interesting aspect with the assumption that a portion of the cell population developed a resistant mechanism to quinoin through the proposed mechanisms as previously reported [33].

According to the IC_50_ value, we evaluated the effect of quinoin on human glioma cells growth rate, applying the drug at concentrations of 2.5 and 5.0 nM each day for a total of 3 days, starting one day after plating.

**Figure 2:**
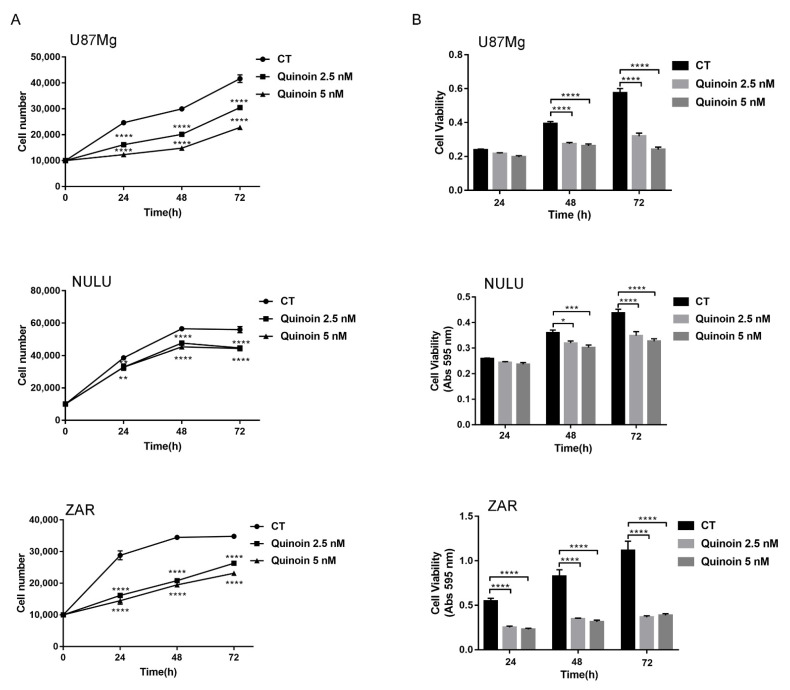
Growth curve and MTT assay of the U87Mg glioblastoma continuous cell line and primary cell lines. (**A**) On the left, the graphs of the growth curves of the continuous glioblastoma cell line U87Mg and of two primary cell lines obtained from the patient’s biopsy (NULU and ZAR) are shown. Quinoin was administered at various doses of 2.5 and 5.0 nM daily, at various time intervals (1, 2, 3 days). (**B**) On the right, the graphs of the cell viability assessed by MTT assay. U87Mg and patient-derived glioblastoma cell lines NULU and ZAR treated daily with quinoin 2.5 and 5.0 nM, at various time intervals (1, 2, 3 days). Data shown are representative of three separate experiments and values are presented as Mean ± SEM. Statistical analysis was performed by one-way ANOVA. According to GraphPad Prism, * *p*-value 0.01 to 0.05 (significant), ** *p*-value 0.001 to 0.01 (very significant), *** *p*-value 0.0001 to 0.001 (extremely significant), **** *p*-value < 0.0001 (extremely significant).

These treatments reduced the linear phase of growth in both the continuous cell line U87Mg and in primary glioblastoma cell lines (NULU and ZAR), with the cell number already being substantially reduced at 1 day after the beginning of the treatment and increased after two and three days (Figure 2A).

The cytotoxicity of quinoin on glioblastoma cells, evaluated by MTT assay, revealed a significant reduction of the cell metabolic activity at concentrations of 2.5 and 5.0 nM. In particular, the primary cell line ZAR proved to be the most sensitive to quinoin treatment among the three glioblastoma cell lines, exhibiting a high response after 24 h of exposure (Figure 2B).

### 2.3. Quinoin Treatment Results in Morphological Alteration in U87Mg Cells

**Figure 3:**
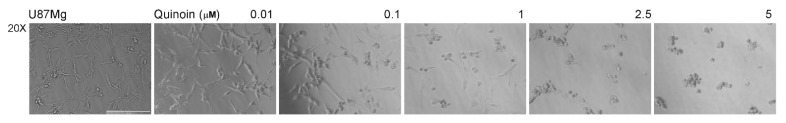
Morphological change of quinoin-treated U87Mg. The glioblastoma continuous cell line was exposed to 0.01, 0.1, 1.0, 2.5, and 5.0 μM quinoin for 72 h. Cells were imaged with an Evos FL microscope at 20× magnification.

After 72 h of quinoin treatment at different concentrations (1.0, 2.5, and 5.0 µM), U87Mg cells revealed dramatic morphological changes by microscopic observation. The cells lost their polygonal shape and filaments, and cell shrinkage occurred to acquire a rounded phenotype typical of apoptotic cells (Figure 3).

The different response to quinoin treatment reflects the heterogeneous phenotype of primary glioblastoma cells.

**Figure 4:**
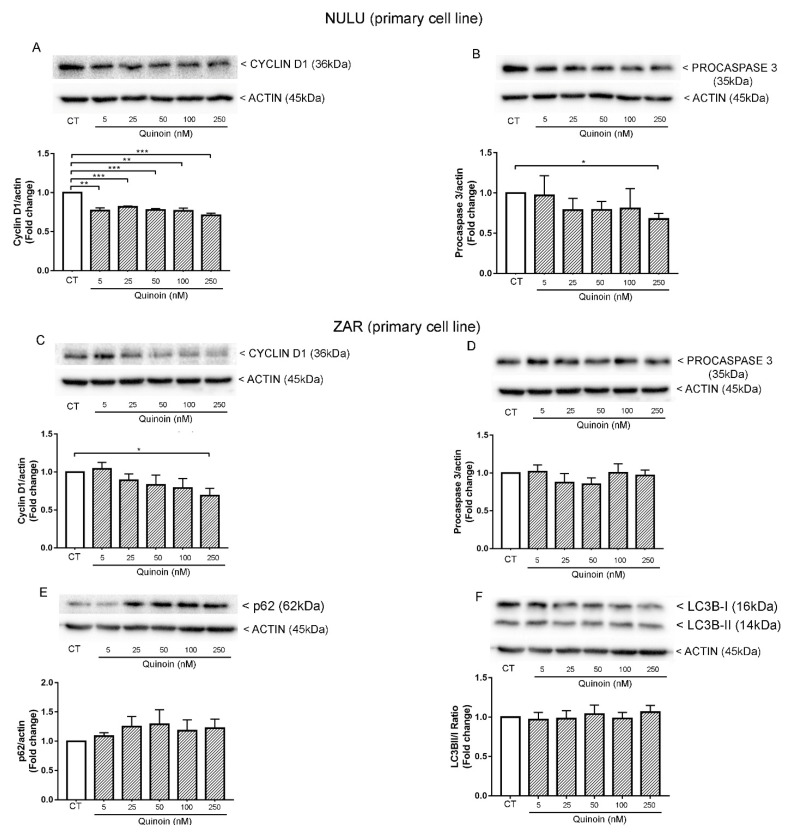
Western blot analysis of quinoin-treated primary cell lines for 24 h. (**A**) Quinoin induced a significant time-independent reduction of Cyclin D1 and (**B**) activation of apoptosis by a decrease of procaspase 3 when administered at a concentration of 250 nM in the NULU cell line. (**C**) Western blot analysis of the expression of Cyclin D1, (**D**) procaspase, and autophagic markers p62 (**E**) and LC3B (**F**) after treatment of the ZAR cell line with different concentrations of quinoin for 24 h. Densitometric analysis of protein levels represent the means ± SEM of three individual determinations. Data were normalized to the housekeeping gene actin and are expressed as a fold change over control-treated cells. * Unpaired *t*-test. According to GraphPad Prism, * *p*-value 0.01 to 0.05 (significant), ** *p*-value 0.001 to 0.01 (very significant), *** *p*-value 0.0001 to 0.001 (extremely significant).

Western blot analysis of patient-derived glioblastoma cells NULU treated with increasing concentrations of quinoin showed a dose-independent reduction of Cyclin D1 (Figure 4A and Figure S2), while the ZAR cell line exhibited a significant reduction of Cyclin D1 at the maximal concentration used (250 nM) (Figure 4C).

Different responses of the primary cell lines to quinoin treatment were also revealed by investigating the activation of the apoptotic pathway. In this regard, the slight reduction of procaspase 3 in the NULU cell line was not detected in the ZAR cell line (Figure 4B,D).

The decrease of procaspase 3 was followed by a contemporary appearance of the activated form, which was visibly increased in the lysates of the cells treated with quinoin (Figure S3).

This heterogeneous response reflects the heterogeneous nature of primary glioblastoma cell lines, which faithfully reproduce the parental tumor from that they are derived [30]. Since the potential arrest of the cell cycle and activation of apoptosis are not the lead mechanisms underlying the quinoin-mediated cytotoxicity in the ZAR cell line, the induction of autophagy was also investigated. However, the common markers of the autophagic pathway, p62 and LC3B, did not show a significant change (Figure 4E,F), leading us to exclude the involvement of autophagy in primary glioblastoma cells treated with quinoin.

### 2.4. Quinoin and Oxidative Stress

**Figure 5:**
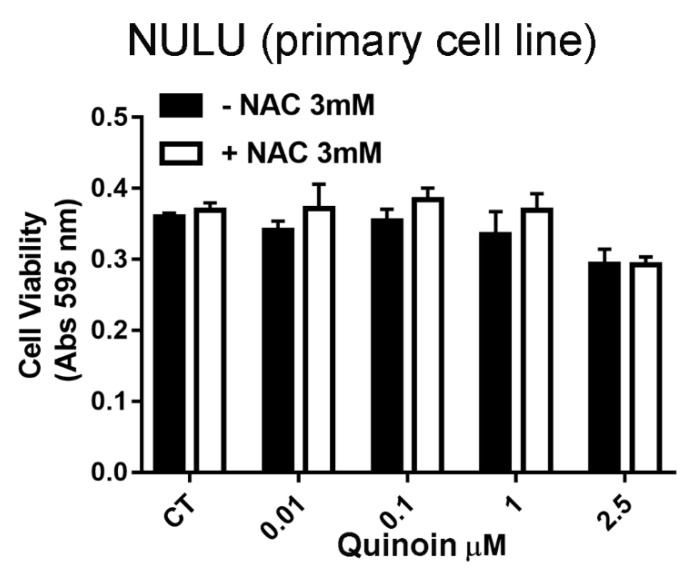
Quinoin and oxidative stress. Effect of primary cell line NULU’s pre-treatment with the ROS scavenger NAC (3.0 mM) and evaluation of the cell viability under different concentrations of quinoin (0.01, 0.1, 1, and 2.5 µM) at 24 h from treatment. Data analyzed with the unpaired *t*-test revealed no significance.

In order to clarify the molecular mechanism of quinoin’s action, the involvement of oxidative stress in quinoin-induced cytotoxicity was investigated. However, pretreatment with the ROS scavenger NAC (3.0 mM) indicated that the cytotoxic effects of quinoin are not mediated by oxidative stress (Figure 5).

### 2.5. Quinoin as a Potential Adjuvant for Glioblastoma Treatment in Combination with Temozolomide

**Figure 6:**
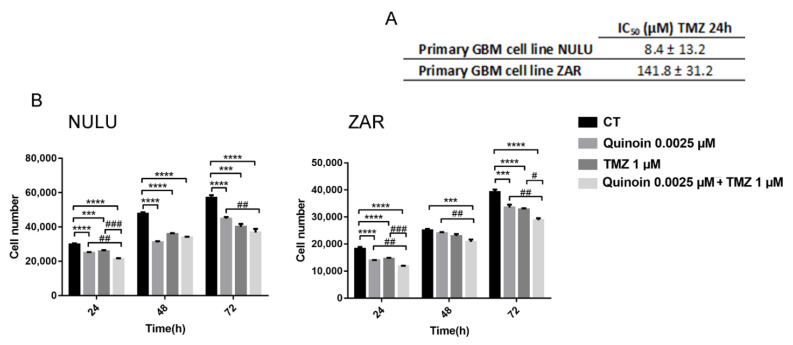
Combined treatment of quinoin and TMZ on primary glioblastoma cell lines. (**A**) IC_50_ values of TMZ of two primary glioblastoma cell lines, NULU and ZAR, after 24 h of incubation with TMZ. Data were processed using GraphPad Prism and data are reported as Mean ± SD. (**B**) Combined treatment in the presence of quinoin 2.5 nM and TMZ 1.0 μM for 24 h on NULU and ZAR primary cell lines. Data shown are representative of three separate experiments and values are presented as Mean ± SEM. Statistical analysis was performed by one-way ANOVA. According to GraphPad Prism, *** *p*-value 0.0001 to 0.001 (extremely significant), **** *p*-value < 0.0001 (extremely significant) significance vs. control cells; # *p*-value 0.01 to 0.05 (significant) significance of TMZ 1 μM vs. quinoin 2.5 nM plus TMZ 1.0 μM; ## *p*-value 0.001 to 0.01 (very significant) significance of quinoin 2.5 nM vs. quinoin 2.5 nM plus TMZ 1 μM; ### *p*-value 0.0001 to 0.001 (extremely significant) significance of TMZ 1 μM vs. quinoin 2.5 nM plus TMZ 1.0 μM.

Although the promoter of the O^6^-methylguanine-methyltransferase (MGMT) gene was previously reported to be unmethylated in the primary cell lines NULU and ZAR [30], thus predicting potential TMZ resistance, the effective sensitivity to TMZ was verified by determining the IC_50_ value at 24 h. NULU, with an IC_50_ value of 8.4 ± 13.2 μM, was found to be more sensitive to TMZ with respect to ZAR (IC_50_ 141.8 ± 31.2 μM, Figure 6A).

The combination of quinoin (2.5 nM) with TMZ (1.0 μM) was found to be efficient in patient-derived GBM cell lines after 24 h of exposure (Figure 6B), indicating the potential of quinoin as a possible adjuvant in the treatment of glioblastoma.

## 3. Discussion

Glioblastoma is the most serious and common brain tumor affecting adults. It is malignant, infiltrating, expansive, and has a rapid growth pathology. These aspects, together with high angiogenesis, cellular heterogeneity, and the presence of a specific population of stem cells (brain tumor stem cells) that can proliferate and generate neoplastic glial cells [34], contribute to a poor prognosis: the median survival for this type of cancer is 14 months [28] with a 5-year survival rate of 2% [35].

There are numerous histopathological variants of GBM. In any case, features common to all types of GBM are cellular and nuclear pleomorphism, microvascular proliferation, and necrosis. In addition, GBM cells have an ability to activate numerous resistance mechanisms, complicating the search for effective therapy for this tumor. Among the pharmaco-resistant mechanisms to the alkylating agent (Temozolomide), the most common one found in GBM is O^6^-methylguanine-methyltransferase (MGMT), a specific DNA repair protein, whose expression is variable due to the acquired methylation of the gene promoter during gliomagenesis. Despite the extensive surgical removal of what appears to be all microscopic diseases, either at the initial diagnosis or at the time of relapse, all patients will continue to show tumor growth and progression due to the rapid proliferation of infiltrative disease that persists after surgery. The current standard of care for the newly diagnosed disease includes maximum safe resection, followed by 6 weeks of concomitant daily radiotherapy and chemotherapy with temozolomide (TMZ) [28]. The addition of TMZ improves overall survival by ~2.5 months compared to radiation alone [29]. Notwithstanding attempts to improve outcomes for the newly diagnosed disease, effective treatment for glioblastoma remains unsolved. In this context, we assayed quinoin, type 1 RIP from quinoa seeds on a U87Mg continuous glioblastoma cell line and NULU and ZAR, two primary glioblastoma cell lines. Indeed, primary glioblastoma cell lines, developed from patients’ biopsies, represent the genetic and histological features of patients [30]. The first experiment to evaluate quinoin IC_50_ revealed that this toxin shows high toxicity when glioblastoma cells were treated for 24, 48, and 72 h with concentrations ranging from 0.01 to 5.0 µM. The toxic effect of quinoin was evaluated on primary cell lines too, revealing the same toxicity profile. Moreover, the IC_50_ for all three glioblastoma cell lines did not decrease over time, indicating that quinoin toxicity is not time dependent. Therefore, we decided to use a single time point of 24 h for further experiments. The treatment with various toxin concentrations determined clear morphological change in U87Mg. Indeed, starting from quinoin 1.0 µM, the cells acquired a round form typical of apoptotic cells to reach a maximum effect at 5.0 µM (Figure 3). At this concentration, cells are completely rounded and without extensions. Moreover, growth curves at 1, 2, and 3 days after treatment with quinoin 2.5 or 5.0 nM displayed a statistically significant growth reduction, already at 1 day of treatment (Figure 2A), while MTT analysis in the same conditions reflected the trend of the growth curves (Figure 2B).

On the other hand, the common conventional GBM cell lines (e.g., U87Mg, U251, T98G, and A172) are genetically far from primary tumors due to the high number of passages in culture [36,37], losing the heterogeneity of GBM cells. Thus, we decided to investigate quinoin’s effect on patient-derived cell lines, which resemble the parental tumor and are commonly used as a GBM model [30].

The analysis by Western blot showed that quinoin reduced the expression levels of Cyclin D1 in patient-derived glioblastoma cells NULU and ZAR, assuming that according to other mechanisms of RIPs action, quinoin induced a cell cycle arrest in G1/S phase [20]. However, considering the heterogeneity of GBM, quinoin induced cleavage of procaspase 3 and thus the action of the apoptotic pathway in the NULU primary cell line but not in the ZAR primary cell line. In the above tested conditions, quinoin induced cell death by reducing the expression levels of Cyclin D1 in NULU, but other possible mechanisms must also be investigated in other cell lines at different concentrations of this toxin. Several authors report another possible mode of action triggered by RIP toxins involving either reactive oxygen species (ROS) or the autophagy pathway. Indeed, some RIPs act by a a mechanism that involves reactive oxygen species (ROS) production in response to stress and increased intracellular calcium levels (e.g., abrin, trichosanthin) while, vice versa, other RIPs induce autophagy (e.g., gelonin, trichosanthin, and elderberry RIPs [17,38,39,40]). In this context, we evaluated the proteins involved in the autophagic process LC3BII/LC3BI and p62, which did not change when glioblastoma cells were treated with quinoin. Furthermore, quinoin cytotoxicity is not mediated by oxidative stress, since the pre-treatment with NAC 3.0 mM did not provide protection from quinoin’s effects. These data reported here represent only the features of an innovative point of view to treat glioblastoma cell lines. The choice to investigate these genes (Cyclin D1, Caspase 3, p62, and LC3B) was suggested on the basis of literature, in which analogue pathways were investigated [20,40,41] for RIP proteins, such as saporin-6, tested on glioblastoma cell lines, GL15 and U87MG. Finally, we analyzed the effect of quinoin on glioblastoma cells’ growth in the presence of canonical TMZ chemotherapy. The two drugs were combined according to the IC_50_ for TMZ and quinoin on both primary cell lines. Figure 6 highlights that in the presence of temodal, quinoin can determine a synergistic effect on both primary cell lines examined (NULU and ZAR), which exhibit a particular pharmaco-resistance due to the MGMT promoter unmethylation status [30]. To characterize the synergism between temodal and quinoin, we decided to test a lower concentration of quinoin (0.0025 µM) and Temodal (1.0 µM), thus making evaluation of the synergistic effects normally expected possible.

The synergistic interactions between the two drugs allow a reduction of the doses of the single drugs, obtaining in any case a complete therapeutic effect. The ability to predict this type of pharmacological function is therefore important because it helps to decrease toxicity and side effects. In this case, quinoin could also overcome drug resistance in glioblastoma cells, as drug resistance is responsible in most cases for the failure of drug therapy and early death of the patient [42]. This gives hope that quinoin could be used as an adjuvant drug at very low concentrations, given the high toxicity of the protein, although therapy with RIPs, capable of damaging protein synthesis in a non-discriminatory manner, cannot be considered without engineering the RIPs to target it only versus cancer cells.

Overall, quinoin exhibits cytotoxic action on both the U87Mg glioblastoma continuous cell line and primary cell lines NULU and ZAR and a synergic effect when used with Temozolomide. On the other hand, like other RIPs, quinoin is likely non-selective, probably presenting important limitations, such as blood-brain barrier impermeability, low rate of cell degeneration, inflammatory response, and activation of compensatory mechanisms that limit the use of RIPs alone [13]. These limitations can be overcome by toxin-conjugates considering their possible use as weapons against different cancer cells. Indeed, several immunotoxins [17] or nanoconstructs [12] were obtained to make RIPs selective.

In this framework, quinoin, similar to saporin-6 [21,24,31], is an attractive archetype of this toxin family and could represent a novel tool in biomedicine. Data reported in this work are the starting point for its possible use in neuroscience and in tumor therapy. Finally, since we cannot completely exclude that quinoin is able to pass through the blood-brain barrier, further experiments will be carried out, considering that the blood-brain barrier is disrupted in patients affected by glioblastoma [43,44].

## 4. Materials and Methods

### 4.1. Materials

Chemicals and chromatography for quinoin purification and the set-up conditions for RP-HPLC and SDS-PAGE analysis were obtained as previously reported [24].

### 4.2. Quinoin Purification

Native quinoin was purified from the seeds of white quinoa (*Chenopodium quinoa* Wild) as previously described [24] using a general protocol for the preparation of type 1 ribosome-inactivating proteins [32]. Determination of the protein concentration was achieved using the BCA colorimetric assay [ThermoFisher Scientific, Rodano (MI), Italy].

### 4.3. Cell Culture

The U87Mg human GBM cell lines were obtained from the Sigma Aldrich Collection (LGC Promochem, Teddington, UK). Cells were grown in Dulbecco’s modified Eagle medium (DMEM) with the addition of 10% fetal bovine serum (FBS), 2 mmol/L-glutamine, 100 IU/mL penicillin, and 100 μg/mL streptomycin at 37 °C, 5% CO_2_, and 95% humidity.

Human glioblastoma primary cell cultures were obtained from bioptic samples surgically removed from patients, who gave informed consent to participate in the study. The use of primary cell lines as a model for GBM heterogeneity was approved by Ethics Committee on 27 February 2020 and registered on ClinicalTrials.gov with the identification number NCT04180046. Samples were labelled using a three-letter code. After mechanical dissociation, single cells were resuspended in DMEM medium and centrifuged at 1200 RPM for 5 min. The pellet was resuspended in DMEM medium supplemented with 10% FBS and cells were plated on Petri plates (Falcon Primaria, Lincoln Park, NJ, USA). The medium was then changed every 3 days. After 14–15 days, cells were trypsinised, and re-plated into 24-well plates at a density of 25 × 10^3^ cells/well. The established patient-derived GBM cell lines have been characterized and the genetic profile was previously reported [30].

### 4.4. IC_50_ Estimation of Quinoin and Temozolomide in U87Mg and Patient-Derived GBM Cell Lines

U87Mg cells and primary cell lines NULU and ZAR were plated in 96 wells with a density of 5 × 10^3^ cells/well. The IC_50_ values of quinoin were determined at 24, 48, and 72 h using the MTT assay at concentrations of 0.01, 0.1, 1.0, 2.5, and 5.0 μM. In the same way, the IC_50_ value after 24 h of conventional chemotherapy with TMZ was determined in patient-derived glioblastoma cells NULU and ZAR, using TMZ concentrations of 10, 50, 100, 150, and 200 μM. Data were processed using GraphPad Prism (GraphPad Software Inc., San Diego, CA, USA) (https://www.graphpad.com/guides/prism/latest/curve-fitting/reg_50_of_what__relative_vs_absolu.htm; accessed on 21 September 2021) assuming the control as part of the dose–response curve, considering it as a very low concentration (10^−11^ µM).

### 4.5. Proliferation Assay

In order to evaluate the response to quinoin, U87Mg and patient-derived GBM cell lines were plated in 48 wells at a density of 1 × 10^4^ cells/well in DMEM with 10% FBS, incubating them at a temperature of 37 °C with 5% of CO_2_. On the basis of the IC_50_ values, cells were then treated daily with quinoin at established concentrations of 2.5 and 5.0 nM for 24, 48, and 72 h. The cell count was then performed using a Burker chamber.

### 4.6. Cell Viability Test

U87Mg and patient-derived GBM cell lines were plated in 96 wells at a density of 5 × 10^3^ cells/well and treated daily for 24, 48, and 72 h at quinoin concentrations of 2.5 and 5.0 nM. The MTT assay (3-(4,5-dimethylthiazol-2-yl)-2,5-diphenyltetrazolium) (Sigma Chemical Co., St. Louis, MO, USA) was then performed. Specifically, 5 mg/mL of MTT were added to 100 μL of cells cultured in DMEM. The formazan crystals were dissolved with 0.4% isopropanol/HCl and the absorbance was measured at 595 nm with a plate reading spectrophotometer. To evaluate whether quinoin caused oxidative stress, primary GBM NULU cells were plated at a density of 5 × 10^3^ cells/well and starved for 48 h in DMEM with 0.5% FBS. Cells were then pre-treated with the antioxidant N-acetylcysteine (NAC) 3.0 mM for 4 h at 37 °C in DMEM with 10% FBS. The medium was replaced, and the cells were treated with quinoin at concentrations of 0.01, 0.1, 1.0, and 2.5 μM.

### 4.7. Microscopic Observation of Live Cells

U87Mg cells were plated in 96 wells in DMEM and 10% FBS and treated with 0.01, 0.1, 1.0, 2.5, and 5.0 μM of quinoin for 72 h. After treatment, the cells were observed with a phase contrast microscope (Evos, Life technologies, Monza, Italy) and morphological changes were evaluated.

### 4.8. Combined Treatment with TMZ and Quinoin

Primary GBM cell lines NULU and ZAR were plated in 48 wells at a density of 1 × 10^4^ cells/well and treated with TMZ 1.0 μM either alone or in combination with quinoin 2.5 nM for 24, 48, and 72 h. After each treatment, the cells were counted through a Burker chamber.

### 4.9. Western Blot Analysis of U87Mg Cells Treated with Quinoin

In order to determine the protein expression in glioblastoma quinoin-treated cells, an extraction was performed with Triton X-100 lysis buffer (10 mM Tris•Cl, 1.0 mM EDTA, 150 mM NaCl, 1% Triton X-100, NaF 1.0 mM, 1.0 mM Na_4_P_2_O_7_, 1.0 mM Na_3_VO_4_ and 1× protease inhibitors). Following the lysis, the extracted proteins (15 μg) were separated by 12.5% SDS-PAGE and transferred to PVDF membranes by electroblotting. The membranes were first saturated and incubated for 1 h at room temperature with 5% non-fat dry milk or BSA (bovine serum albumin) diluted in Tris 1× buffered saline containing Tween-20 (TBST), and subsequently incubated with specific primary antibodies overnight at 4 °C. Each membrane was also incubated with mouse monoclonal anti-β-actin (1: 10,000, Santa Cruz Biotechnology, Dallas, TX, USA) for protein normalization. The membranes were then exposed to secondary antibodies conjugated with the HRP enzyme (Calbiochem, Merk Life Science Srl., Milan, Italy). The protein bands were visualized by the chemiluminescence process using ECL Western blotting (GE healthcare Life Sciences, Milan, Italy), while the digital signals were quantified by densitometric analysis using the Image Lab software (Bio-Rad Laboratories, Rome, Italy).

To monitor the expression of the cell cycle protein Cyclin D1, the apoptotic protein procaspase 3, and the autophagic proteins p62 and LC3B, cells were plated at a density of 5 × 10^5^ cells in 60 mm plates in DMEM (without FBS) and incubated for 48 h. After adding 10% FBS, cells were treated with different concentrations of quinoin (5.0, 25, 50,100, and 250 nM) for 24 h. The membranes were incubated with the antibodies anti-p62 and anti-LC3B (1:1000, Cell Signaling Technology, Euroclone, Pero, Italy), anti-Caspase 3 (1:1000, Cell Signaling Technology), and anti-Cyclin D1 (1:1000, Cell Signaling Technology).

### 4.10. Statistical Analysis

Data obtained from the experiments performed in triplicate are expressed as mean ± SEM and were analyzed by Student’s-*t* test or one-way ANOVA. The differences were considered significant for *p* < 0.05. Analyses were carried out using the GraphPad Prism (GraphPad Software Inc.).

## 5. Conclusions

Until now, the effective treatment of glioblastoma disease represents one of the most important challenges for researchers. Moreover, poor prognosis, mainly due to therapeutic resistance and tumor recurrence after surgical removal, as well as tumor heterogeneity, have complicated the search for an effective glioblastoma therapy. On the other hand, the chemotherapeutic agent TMZ, used in current glioblastoma treatment, improves overall survival, although it does not provide an effective treatment for the disease. In this context, we investigated the cytotoxicity of quinoin, a novel type 1 RIP from quinoa seeds, on human continuous and primary glioblastoma cell lines while also evaluating the effect of this toxin towards primary cells, also in combination with TMZ. Interestingly, these findings suggest that RIPs could represent a novel effective strategy for glioblastoma therapy and a possible adjuvant for the treatment of the disease in combination with TMZ.

Overall, this study reveals that quinoin is a novel attractive tool in glioblastoma research that can counteract the growth of these cancer cells. In addition, the synergic effect of this toxin with canonical chemotherapy opens the way to possible uses of this toxin in strategies providing for its use in immunoconjugates or nanoconstructs to minimize the adverse effects in vivo when this toxin is used alone.

## Data Availability

The data presented in this study are available on request from the corresponding author.

## Supplementary Materials

The following are available online at https://www.mdpi.com/article/10.3390/toxins13100684/s1, Figure S1: (A) SDS-PAGE analysis of the purified quinoin with or without β-mercaptoethanol (lanes 1–2 and 3–4; 3.0 and 6.0 μg, respectively; M, protein markers). SDS-PAGE was carried out in 12% polyacrylamide separating gel. (B) Elution profile of purified quinoin by RP-HPLC. Quinoin (100 μg) was separated on a C4 column (Phenomenex, 0.46 × 25 cm), using a Waters Breeze HPLC system. The elution system contained 0.1% trifluoroacetic acid (TFA) in H_2_O (solvent A) and 0.1% TFA in acetonitrile (solvent B). A gradient elution system was applied from 5% to 65% of solvent B in 60 min at a flow rate of 1.0 mL/min. Figure S2: Western Blot analysis of Cyclin D1 in primary glioblastoma cell lines NULU (A) and ZAR (B) treated with Quinoin 2.5 nM for 24, 48, and 72 h. Figure S3: Western blot analysis of Caspase 3 in primary glioblastoma cells NULU treated with Quinoin 5, 25, 50, 100, and 250 nM for 24 h. The blot revealed the presence of activated form of caspase 3.
