# Supplementary material for: Cytotoxicity Effect of Quinoin, Type 1 Ribosome-Inactivating Protein from Quinoa Seeds, on Glioblastoma Cells
Source: Toxins (Basel). 2021 Sep 25;13(10):684. doi: 10.3390/toxins13100684 (PMC8537469; doi:10.3390/toxins13100684)
Supplement: Supplementary file 1 [file toxins-13-00684-s001.zip › toxins-1352048-supplementary.pdf]

toxins
MDPI

*Article*

# Cytotoxicity Effect of Quinoin, Type 1 Ribosome-Inactivating Protein from Quinoa Seeds, on Glioblastoma Cells

**Rossella Rotondo [1,†], Sara Ragucci [2,†], Salvatore Castaldo [1], Maria Antonietta Oliva [1], Nicola Landi [2], Paolo V. Pedone [2], Antonietta Arcella [1,*,‡] and Antimo Di Maro [2,‡]**

1 INM IRCCS Istituto Neurologico Mediterraneo NEUROMED, Via Atinense 18, 86077 Pozzilli, Italy; rossellaross1988@gmail.com (R.R.); castaldosal90@gmail.com (S.C.); mariaantonietta.oliva@neuromed.it (M.A.O.)
2 Department of Environmental, Biological and Pharmaceutical Sciences and Technologies (DiSTABiF), University of Campania "Luigi Vanvitelli", Via Vivaldi 43, 81100 Caserta, Italy; sara.ragucci@unicampania.it (S.R.); nicola.landi@unicampania.it (N.L.); paolovincenzo.pedone@unicampania.it (P.V.P.); antimo.dimaro@unicampania.it (A.D.M.)
* Correspondence: arcella@neuromed.it
† These authors contributed equally to this work.
‡ These authors share equal senior authorship.

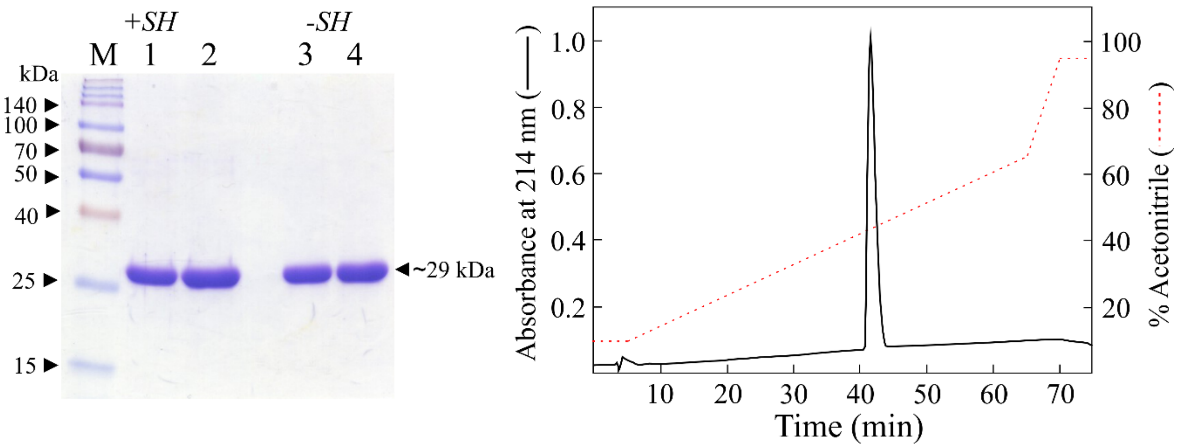


**Figure S1.** (A) SDS-PAGE analysis of the purified quinoin with or without β-Mercaptoethanol (lanes 1-2 and 3-4; 3.0 and 6.0 μg, respectively; M, protein markers). SDS-PAGE was carried out in 12% polyacrylamide separating gel. (B) Elution profile of purified quinoin by RP-HPLC. Quinoin (100 μg) was separated on a C4 column (Phenomenex, 0.46x25 cm), using a Waters Breeze HPLC system. The elution system contained 0.1% trifluoroacetic acid (TFA) in H2O (solvent A) and 0.1% TFA in acetonitrile (solvent B). A gradient elution system was applied from 5% to 65% of solvent B in 60 minutes at a flow rate of 1 mL/min.

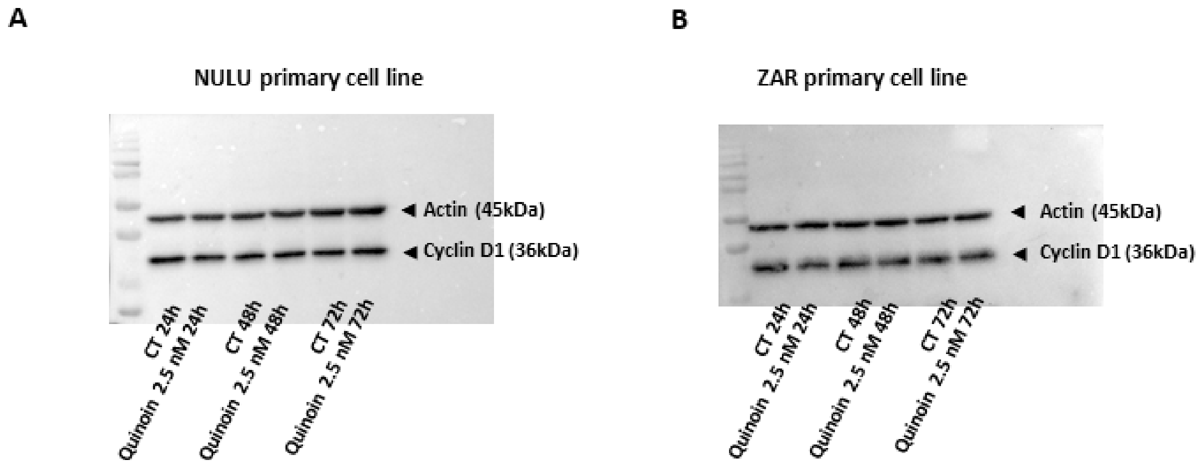


**Figure S2.** Western Blot analysis of Cyclin D1 in primary glioblastoma cell lines NULU (A) and ZAR (B) treated with Quinoin 2.5 nM for 24, 48 and 72 h.

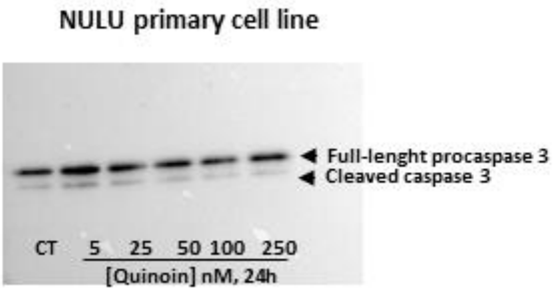


**Figure S3.** Western Blot analysis of Caspase 3 in primary glioblastoma cells NULU treated with Quinoin 5, 25, 50, 100 and 250 nM for 24 h. The blot revealed the presence of activated form of caspase 3.
